# Supplementary material for: Persistent epigenetic memory of SARS-CoV-2 mRNA vaccination in monocyte-derived macrophages
Source: Mol Syst Biol. 2025 Mar 25;21(4):2. doi: 10.1038/s44320-025-00093-6 (PMC11965535; doi:10.1038/s44320-025-00093-6)
Supplement: Supplementary file 14 — Expanded View Figures [file 44320_2025_93_MOESM14_ESM.pdf]

Expanded View Figures

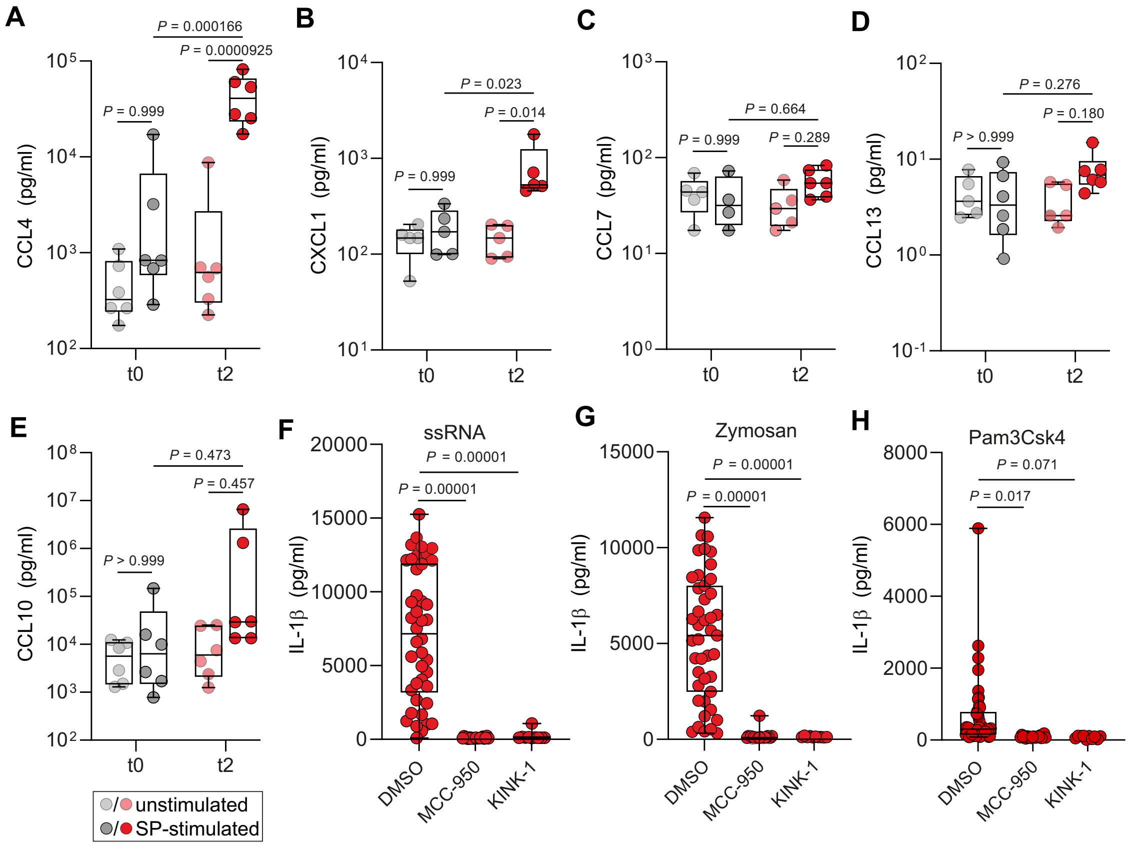

Figure EV1. Inflammasome dependent secretion of IL-1β in macrophages following vaccination.

(**A**) Monocytes were isolated by CD14+ selection from blood samples from unvaccinated (t0) ($n = 6$) (gray) and vaccinated individuals (t2) ($n = 6$) (red) and seeded and incubated in the presence of M-CSF for 5 days. Differentiated macrophages were stimulated with SP (t0: dark gray dots; t2: dark red dots) or left unstimulated (t0: light gray dots; t2: light red dots). Concentrations of CCL4, CXCL1 (**B**), CCL7 (**C**), CXCL13 (**D**) and CXCL10 (**E**), were measured by multiplex analyses. For statistical analysis, two-way ANOVA with Sidak's multiple comparison analysis was used. Box plots indicate the median, the upper and lower quartile and the minimum and maximum values. (**F**) Monocyte-derived macrophages from donors before (t0) and 2 weeks after second vaccination (t2) were generated as described in (**A**). Differentiated macrophages were stimulated with ssRNA, Zymosan (**G**) or Pam_3Csk_4 (**H**) for 4 h in the presence of MCC950 (10 μM) ($n = 20$), KINK-1 (5 μM) ($n = 10$) or left untreated. IL-1β secretion was quantified by ELISA. For statistical analysis, one-way ANOVA with Dunnett's multiple comparison test comparing MCC950/KINK-1-treated cells to untreated cells was used. Box plots indicate the median, the upper and lower quartile and the minimum and maximum values. Shown data points represent the technical mean of an independent experiment. *P* values less than 0.05 were considered statistically significant.

 © The Author(s)

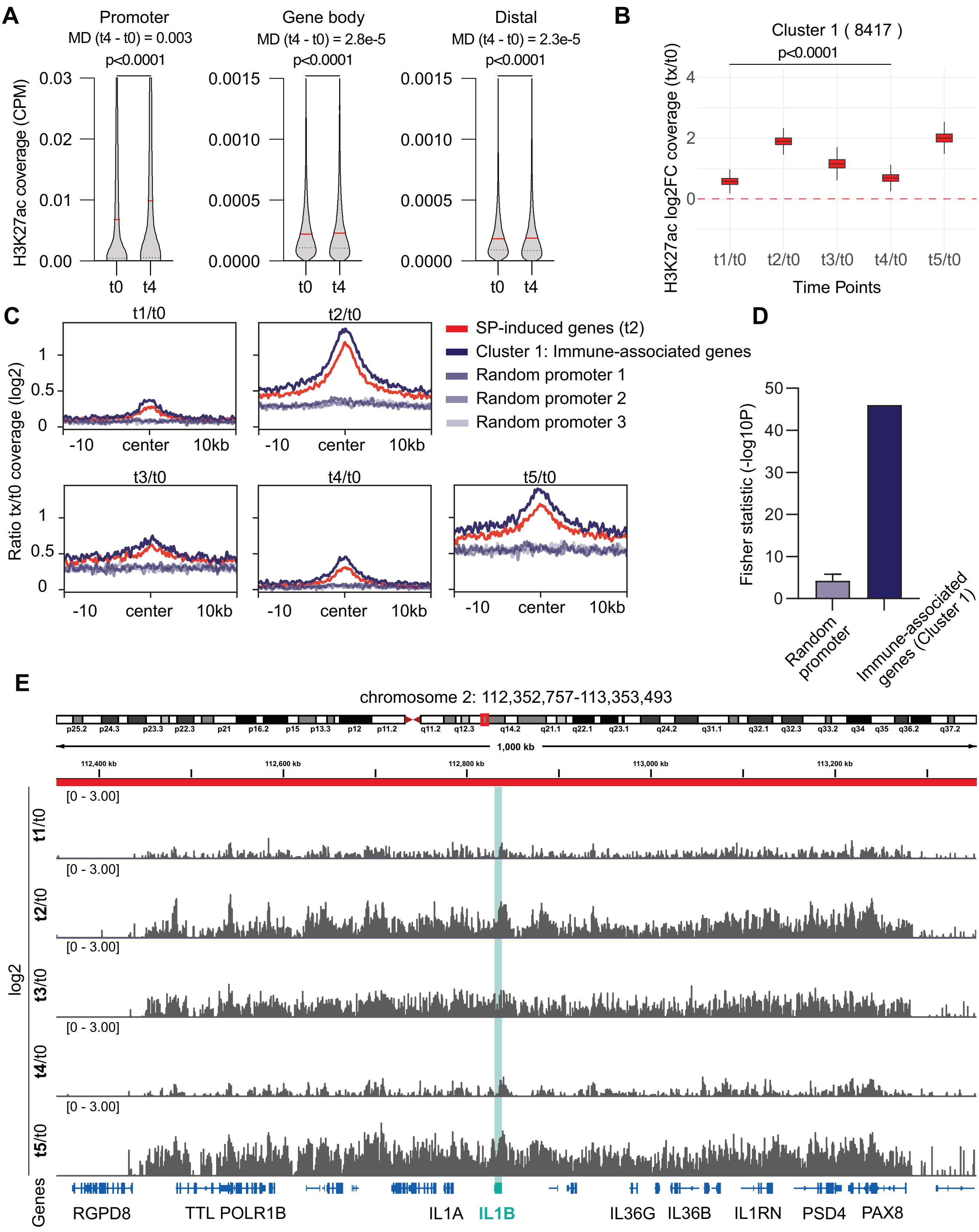
A
Promoter
MD (t4 - t0) = 0.003
p<0.0001
Gene body
MD (t4 - t0) = 2.8e-5
p<0.0001
Distal
MD (t4 - t0) = 2.3e-5
p<0.0001
H3K27ac coverage (CPM)
t0
t4
B
Cluster 1 (8417)
p<0.0001
H3K27ac log2FC coverage (tx/t0)
t1/t0
t2/t0
t3/t0
t4/t0
t5/t0
Time Points
C
SP-induced genes (t2)
Cluster 1: Immune-associated genes
Random promoter 1
Random promoter 2
Random promoter 3
Ratio tx/t0 coverage (log2)
-10
center
10kb
D
Fisher statistic (-log10P)
Random promoter
Immune-associated genes (Cluster 1)
E
chromosome 2: 112,352,757-113,353,493
1,000 kb
[0 - 3.00]
log2
Genes
RGPD8
TTL POLR1B
IL1A
IL1B
IL36G
IL36B
IL1RN
PSD4
PAX8

Figure EV2. H3K27ac coverage across various gene regions and clusters.

(**A**) Distribution of normalized (counts per million) H3K27ac coverage in peaks of promoters (1 kb upstream to 250 bp downstream of transcription start sites [TSS]) (left), gene bodies (251 bp downstream of TSS to transcription end sites [TES]) (middle) and distal regions (1 kb to 100 kb away from TSS and TES) (right). All distribution comparisons are significantly different ($P < 0.0001$). P values were calculated using a Wilcoxon test. P values less than 0.05 were considered statistically significant. Median difference (MD) between t4 and t0 is indicated for all three genomic regions. (**B**) H3K27ac coverage shown as fold change (log2) across time points (t1-t5) relative to t0 for all genes part of Cluster 1. (**C**) Fold change of H3K27ac read coverage in tx relative to t0 in H3K27ac peaks overlapping with selected gene promoter from SP-stimulated genes ($n = 576$), Cluster 1 immune-associated genes ($n = 333$), Random promoter 1 ($n = 333$), Random promoter 2 ($n = 333$), Random promoter 3 ($n = 333$). (**D**) Fisher statistic (−log10P) of H3K27ac peaks in SP-induced gene promoter overlapping with H3K27ac peaks in Cluster 1 immune gene promoter or H3K27ac peaks in random promoter. Error bar indicates standard deviation of the fisher statistic from the 3 randomly distributed H3K27ac peaks in all annotated promoters. (**E**) Genome browser view of 1000 kb of chromosome 2, displaying ratio (log2) of tx over t0 mean H3K27ac coverage across the time points.

 © The Author(s)

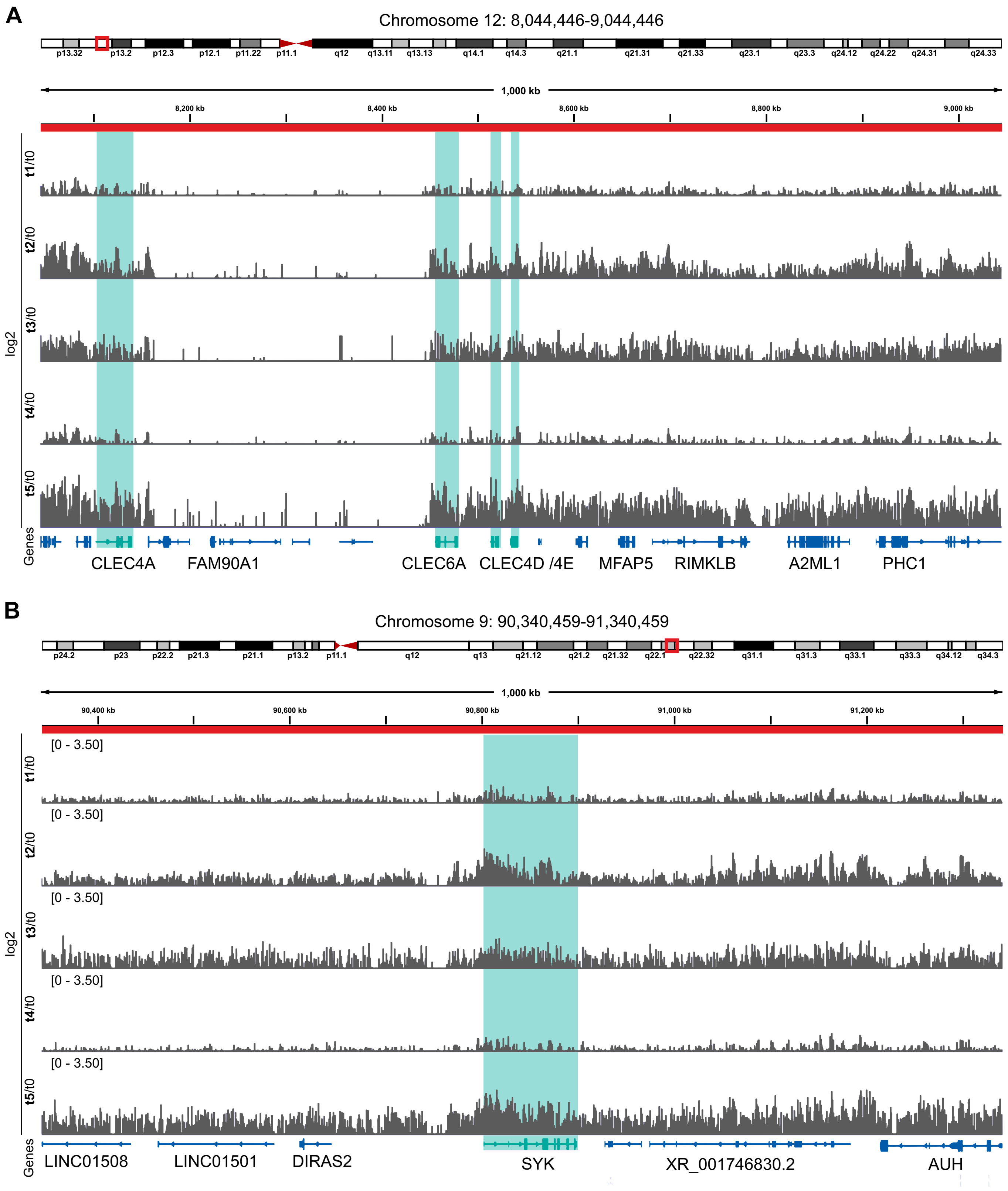

Figure EV3. H3K27ac coverage across different vaccination time points.

(**A**) Genome browser view of 1000 kb of chromosome 12 and 9 (**B**), displaying ratio (log2) of tx over t0 mean H3K27ac coverage across the time points.

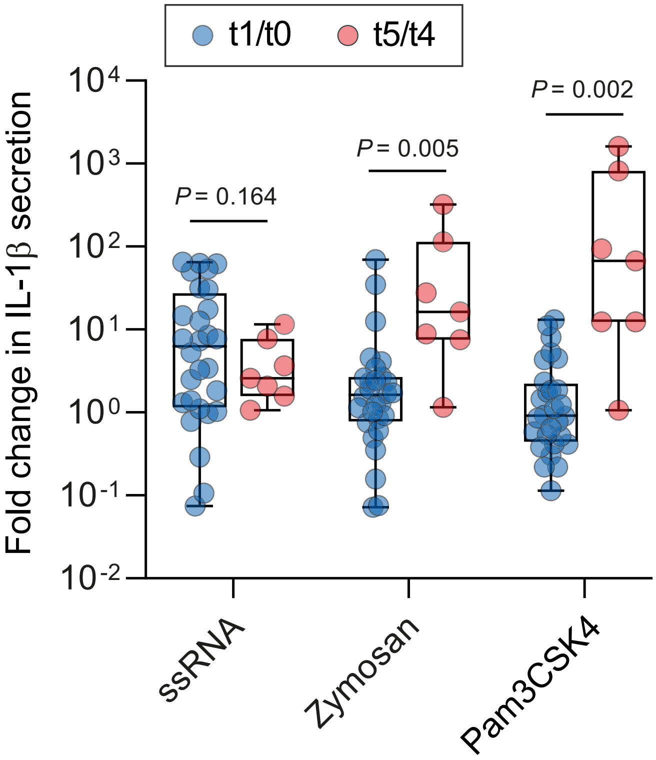

Figure EV4. Cytokine release before and after the third dose booster vaccination.

Fold change of L-1β release upon stimulation with ssRNA, zymosan or Pam3CSK4 between t1/t0 ($n = 28$) (blue) and t5/t4 ($n = 7$) (red). For statistical analysis, a multiple unpaired t test was used. Box plots indicate the median, the upper and lower quartile and the minimum and maximum values. Shown data points represent the technical mean of an independent experiment.

 © The Author(s)
